# Ocean-wide comparisons of mesopelagic planktonic community structures

**DOI:** 10.1038/s43705-023-00279-9

**Published:** 2023-08-18

**Authors:** Janaina Rigonato, Marko Budinich, Alejandro A. Murillo, Manoela C. Brandão, Juan J. Pierella Karlusich, Yawouvi Dodji Soviadan, Ann C. Gregory, Hisashi Endo, Florian Kokoszka, Dean Vik, Nicolas Henry, Paul Frémont, Karine Labadie, Ahmed A. Zayed, Céline Dimier, Marc Picheral, Sarah Searson, Julie Poulain, Stefanie Kandels, Stéphane Pesant, Eric Karsenti, Tara Oceans coordinators, Stefanie Kandels, Stéphane Pesant, Eric Karsenti, Silvia G. Acinas, Emmanuel Boss, Guy Cochrane, Colomban de Vargas, Gabriel Gorsky, Nigel Grimsley, Lionel Guidi, Pascal Hingamp, Lee Karp-Boss, Fabrice Not, Jeroen Raes, Christian Sardet, Sabrina Speich, Peer Bork, Chris Bowler, Daniele Iudicone, Hiroyuki Ogata, Lars Stemmann, Matthew B. Sullivan, Shinichi Sunagawa, Patrick Wincker, Olivier Jaillon, Peer Bork, Chris Bowler, Colomban de Vargas, Damien Eveillard, Marion Gehlen, Daniele Iudicone, Fabien Lombard, Hiroyuki Ogata, Lars Stemmann, Matthew B. Sullivan, Shinichi Sunagawa, Patrick Wincker, Samuel Chaffron, Olivier Jaillon

**Affiliations:** 1 Génomique Métabolique, Genoscope, Institut de Biologie François Jacob, Commissariat à l’Energie Atomique (CEA), CNRS, Université Evry, Université Paris-Saclay, 91000 Evry, France; 2 Research Federation for the study of Global Ocean Systems Ecology and Evolution, FR2022/Tara Oceans GOSEE, 3 rue Michel-Ange, 75016 Paris, France; 3 Sorbonne Université, CNRS, Station Biologique de Roscoff, AD2M, UMR 7144, 29680 Roscoff, France; 4 Nantes Université, École Centrale Nantes, CNRS, LS2N, UMR 6004, F-44000 Nantes, France; 5 Structural and Computational Biology, European Molecular Biology Laboratory, Meyerhofstr. 1, 69117 Heidelberg, Germany; 6 Sorbonne Université, CNRS, Institut de la Mer de Villefranche sur mer, Laboratoire d’Océanographie de Villefranche, 06230 Villefranche-sur-Mer, France; 7 Institut de Biologie de l’ENS (IBENS), Département de biologie, Ecole normale supérieure, CNRS, INSERM, Université PSL, 75005 Paris, France; 8 Department of Microbiology, The Ohio State University, Columbus, OH 43214 USA; 9 Bioinformatics Center, Institute for Chemical Research Kyoto University, Gokasho, Uji, Kyoto, 611-0011 Japan; 10 Stazione Zoologica Anton Dohrn, Villa Comunale, 80121 Naples, Italy; 11 Directors’ Research European Molecular Biology Laboratory Meyerhofstr. 1, 69117 Heidelberg, Germany; 12 MARUM, Center for Marine Environmental Sciences, University of Bremen, Bremen, Germany; 13 PANGAEA, Data Publisher for Earth and Environmental Science, University of Bremen, Bremen, Germany; 14 Department of Bioinformatics, Biocenter, University of Würzburg, Würzburg, Germany; 15 Institut Pierre Simon Laplace, Laboratoire des Sciences du Climat et de l’Environnement, CEA, CNRS, Université Paris-Saclay, 91191 Gif-sur-Yvette cedex, France; 16 Department of Civil, Environmental and Geodetic Engineering, The Ohio State University, Columbus, OH 43214 USA; 17 Department of Biology; Institute of Microbiology and Swiss Institute of Bioinformatics, ETH Zurich, Zurich, 8093 Switzerland; 18 Department of Marine Biology and Oceanography, Institut de Ciències del Mar (ICM), CSIC, Barcelona, Spain; 19 School of Marine Sciences, University of Maine, Orono, ME USA; 20 CNRS, UMR 7232, BIOM, Avenue Pierre Fabre, Banyuls‐sur‐Mer, France; 21 Aix Marseille Univ., Université de Toulon, CNRS, IRD, MIO UM 110, 13288 Marseille, France; 22 Department of Microbiology and Immunology, Rega Institute, KU Leuven Leuven, Belgium; 23 Laboratoire de Physique des Océans, UBO‐IUEM, Place Copernic, Plouzané, France

**Keywords:** Microbial ecology, Community ecology, Community ecology

## Abstract

For decades, marine plankton have been investigated for their capacity to modulate biogeochemical cycles and provide fishery resources. Between the sunlit (epipelagic) layer and the deep dark waters, lies a vast and heterogeneous part of the ocean: the mesopelagic zone. How plankton composition is shaped by environment has been well-explored in the epipelagic but much less in the mesopelagic ocean. Here, we conducted comparative analyses of trans-kingdom community assemblages thriving in the mesopelagic oxygen minimum zone (OMZ), mesopelagic oxic, and their epipelagic counterparts. We identified nine distinct types of intermediate water masses that correlate with variation in mesopelagic community composition. Furthermore, oxygen, NO_3_^−^ and particle flux together appeared as the main drivers governing these communities. Novel taxonomic signatures emerged from OMZ while a global co-occurrence network analysis showed that about 70% of the abundance of mesopelagic plankton groups is organized into three community modules. One module gathers prokaryotes, pico-eukaryotes and Nucleo-Cytoplasmic Large DNA Viruses (NCLDV) from oxic regions, and the two other modules are enriched in OMZ prokaryotes and OMZ pico-eukaryotes, respectively. We hypothesize that OMZ conditions led to a diversification of ecological niches, and thus communities, due to selective pressure from limited resources. Our study further clarifies the interplay between environmental factors in the mesopelagic oxic and OMZ, and the compositional features of communities.

## Introduction

Below the ocean’s sunlit layer lies the mesopelagic zone that occupies around 20% of the global ocean volume [1]. The mesopelagic zone is biologically defined as starting where photosynthesis no longer occurs (<1% irradiance; around 200 m depth), down to its lower boundary where there is no detectable sunlight (around 1000 m depth) [2]. This twilight ecosystem cannot rely on photoautotrophy, but sustains its energetic requirements by the combination of heterotrophic, chemoautotrophic, and chemo-mixotrophic metabolisms, together with physicochemical processes. Among the latter, the fraction of upper ocean productivity that escapes epipelagic recycling and sinks by gravity or is delivered by the daily vertical migration of zooplankton constitutes an essential energy source in deep waters and is a vector for attached organisms [3].

Considerable attention has been devoted to the mesopelagic layer in recent years, given its recognized potential for exploitation for bioresources and fisheries [4], potentially becoming an important source of goods for the global bioeconomy [5]. So far, efforts have been made to increase the knowledge of mesopelagic macrofauna by studying the abundance and diversity of nekton. Concerning the mesopelagic community’s microscopic fraction, previous reports have shown a stratification of planktonic communities by the water column. In this regard, the mesopelagic zone displays a distinct assemblage of dsDNA viruses [6], Nucleo-Cytoplasmic Large DNA viruses [7], prokaryotes [8–10], and eukaryotes [11]. These studies have highlighted a global organization different from that of the surface. For example, the mesopelagic plankton diversity does not show a latitudinal diversity gradient trend from pole-to-pole, peaking at lower latitudes [12], and also displays a higher heterogeneity compared to epipelagic waters [13]. Conversely, the mesopelagic microbiome seems to make crucial links in the food web between phototrophic primary production from the sunlit layer and dark ocean specialized consumers [10, 14, 15].

Among the studies conducted in mesopelagic zones, particular efforts have been made to explore regions characterized by extreme conditions, such as oxygen minimum zones (OMZs). These zones are formed by relatively old, slowly upwelling waters, often lying below highly productive surface zones [16], and are currently increasing in volume in the ocean [17]. OMZ prokaryotic communities are well documented and taxa such as *Nitrospira*, *Marinimicrobia*, and anammox bacteria from the phylum Planctomycetes have been reported as typical taxonomic features for OMZ regions studied so far [18–23]. In contrast, knowledge about viruses and eukaryotic diversity in OMZs is still rudimentary. A prevalence of specific eukaryotic taxa such as Ciliophora, Dinoflagellata, MALV, and Acantharia has been reported, together with a higher metabolic activity of these taxa [24–26]. Viruses may have a key role in OMZ ecosystem feedback by modulating the local community (host-virus relationship) [27–29].

The last decades have seen a significant increase in large-scale oceanic surveys [30–32]. Despite the advances reported in previous studies [10, 33, 34], most mesopelagic community studies have been limited to geographically or ecologically fragmented regions, or to specific taxonomic groups, mainly because of the inherent difficulties of accessing this zone on a global scale [35]. Moreover, the combination of biotic and abiotic factors influencing community structure [36, 37], has been poorly explored in the mesopelagic zone. Extending this knowledge by including more comprehensive and homogeneous datasets from assorted geographical and oceanographic systems should lead to a global understanding of this layer. Understanding plankton community structure and dynamics is fundamental to anticipate the impact of global warming and acidification in these regions.

Here, we present a trans-kingdom omics-based comparative study of epipelagic, oxic- and OMZ-mesopelagic communities. To this end, we compiled *Tara* Oceans survey taxonomic DNA barcodes data from four oceanographic basins using standardized sampling protocols. In particular, we focused on mesopelagic environmental drivers, ecology, and taxa associations networks in both oxic and OMZ.

## Materials and methods

### Sample collection and pre-processing

**Fig. 1:**
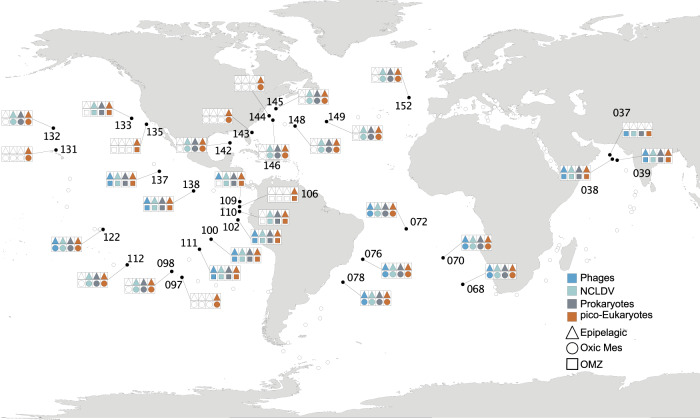
Geographical locations of *Tara* Oceans epipelagic and mesopelagic sampling sites included in this study. Symbol colors represent organism groups evaluated in the present study. Shape formats represent eco-regions, Epipelagic, Oxic MES = oxicmesopelagic, OMZ = oxygen minimum zone mesopelagic.

The environmental and biological data were obtained during the *Tara* Oceans expedition (2009–2012) in 32 oceanographic stations located in the Indian Ocean (IO - 037, 038, 039), Pacific Ocean (PO - 097, 098, 100, 102, 106, 109, 110, 111, 112, 122, 131, 132, 133, 135, 137, 138), South Atlantic Ocean (SAO - 068, 070, 072, 076, 078) and North Atlantic Ocean (NAO - 142, 143, 144, 145, 146, 148, 149, 152) comprising tropical and subtropical regions (Fig. 1). Physico-chemical environmental data were obtained along a vertical profile at each station. Temperature, salinity, and oxygen were measured using a CTD-rosette system with a coupled dissolved oxygen sensor. Chlorophyll-*a* concentrations were measured using high-performance liquid chromatography. Nutrient concentrations were determined using segmented flow analysis. All these metadata are available at PANGAEA [38–43] (10.1594/PANGAEA.875582).

The vertical distribution of marine particles was investigated with an Underwater Vision Profiler (UVP [44, 45],) mounted on the CTD-Rosette. The UVP acquires images in a coherent water volume (1L) delimited by a light sheet issued from red light-emitting diodes. Automatic identification of objects was made using Ecotaxa [46], based on a learning set of visually identified, manually classified objects and associated features. Images were classified to distinguish mesozooplankton from non-living objects and artifacts (e.g., detrital particles, fibers, and out-of-focus objects).

Water vertical profiles of temperature and salinity generated from the CTD were used to identify the water masses by plotting a temperature x salinity (T/S) diagram using the Ocean Data View V 5.0 (ODV) software package [47].

Three different water layers were sampled: surface (SRF, 3–7 m), deep chlorophyll maximum (DCM - depth identified according to the peak of chlorophyll-*a* fluorescence obtained *in situ*), and mesopelagic (ranging from 200–1000 m) [48]. The planktonic community was sampled by partitioning the pumped seawater by filtering each sampled depth with different filter sizes, the on-board sampling methodology is detailed by Pesant et al. [42].

Among the sampled mesopelagic zones, 13 of them were identified as deficient in oxygen and classified as oxygen minimum zone (OMZ, stations IO - 037, 038, 039 / PO - 100, 102, 106, 109, 110, 111, 133, 135, 137, 138). The OMZ were categorized as suboxic: <10 μM O_2_/kg seawater and anoxic: (<0.003 μM/kg seawater or undetectable with most sensitive techniques, e.g., STOX sensors) [Units of O_2_ concentration: 1 mL.L^−1^ = 1.43 mg. L^−1^ or 1 mL.L^−1^ = 44.64 μM] [25].

Our dataset comprises different organismal size-fractions from viruses (two dsDNA-virus bacteriophage families *Podoviridae* and *Myoviridae* - hereafter named as phages and Nucleo-Cytoplasmic Large DNA viruses - hereafter named as NCLDV) to pico-eukaryotes.

Phage libraries were constructed from seawater samples filtered at 0.22 μm, concentrated using iron chloride flocculation, and treated with deoxyribonuclease (DNase). NCLDV *polB* and prokaryotic 16S rRNA gene sequences were extracted from plankton metagenomes sequenced from 0.22–1.6 or 0.22–3 μm filters, and the pico-eukaryote dataset was obtained by V9-18S rRNA gene marker amplification from 0.8–3 or 0.8–5 μm filters. Details of sample preparation and sequencing procedures are fully described in Alberti et al. [49].

Phage read counts was accessed through the search for the marker genes *gp23* (*Myoviridae*) and *polA* (*Podoviridae*) in the protein collection GOV2.0 derived from metagenomic sequencing described in Gregory et al. [6]. The NCLDV read counts profile was obtained from the *polB* marker gene gathered from the OM-RGC.v2 catalog [9] as described in Endo et al. [7]. The Prokaryotic read counts was assessed from a metagenomic dataset called 16S mitag, as described in Sunagawa et al. [8], which does not rely on PCR amplification [50]. Sequences matching “Eukaryota”, “chloroplasts”, and “mitochondria” were removed from the final Prokaryotic OTU table. Clustering and annotation of pico-eukaryote V9-18S rRNA gene PCR-amplicons are described in de Vargas et al. [51], and functional annotation of taxonomically assigned V9-18S rRNA gene metabarcodes was improved afterwards; in this case, we conserved in the final data only sequences assigned to the “Eukaryota” domain. More details concerning the acquisition and pre-processing of the sequence data used in the present study were compiled from earlier publications and are provided in Supplementary Methods. Throughout the manuscript, we use the classical term “OTU” to denote taxonomic units, although we did not employ the clustering technique historically associated with this acronym. More comments about “OTU” usage are available in Supplementary Methods.

To simplify the biological information and obtain a concise epipelagic dataset (EPI), we merged redundant SRF and DCM OTUs by summing their read counts for each taxonomic group and preserved non-redundant OTUs. Putative biases produced by this merging procedure were tested and discarded as shown in the Supplementary Methods; they did not affect the conclusions.

Afterward, to deal with the compositional nature of the data, OTU count matrices (EPI and MES) were transformed using robust CLR (centered log ratio) after adding the value of one as a pseudo count. Robust CLR transformation considers only values greater than 0 to calculate the geometric mean and avoid biases due to sparse data [52].

### Epipelagic and mesopelagic community and environmental differences

We applied an NMDS analysis based on the Bray–Curtis dissimilarity matrix on CLR transformed data. The ‘metaNMDS’ function from the vegan R package [53] was applied to confirm community differences between epipelagic and mesopelagic layers. Homogeneity of the sampled environmental parameters was checked using the ‘betadisper’ function (homogeneity of multivariate dispersions in the vegan package). The analysis was conducted using the Euclidean distance matrix of the environmental variables with the depths (epipelagic, mesopelagic) as group factor. A permutation test statistically confirmed the results.

### Ecological inferences and statistics

Ecological patterns were inferred using environmental variables to constrain the variation observed in biological data (CLR transformed) for planktonic samples using Canonical Correspondence Analysis (CCA) in the vegan R package. A set of physico-chemical variables for the discrete depths were selected for the ecological inferences, such as nitrate (NO_3_^−^), oxygen, temperature, salinity, density, and particles using particle flux UVP data. In order to avoid collinearity among factors, the selected variables were checked for variance inflation factor using the vif.cca function and tested for significance by ANOVA-like tests performed by ‘anova’ implemented in vegan with 999 permutations. The significance of the effect of each variable was tested individually using all others parameters as covariables (independently from their order in the model) by applying the option ‘margin’ to the ‘anova’ function in vegan.

Permutational multivariate analysis of variance (PERMANOVA) was performed with the function ‘adonis’ in vegan to determine the relationship between mesopelagic community composition and predefined water masses based on 999 permutations.

### Classification of organisms eco-region

In order to detect organisms specific to epipelagic (EPI), oxic mesopelagic (Oxic MES), and OMZ eco-regions, only sampling sites containing both epipelagic and mesopelagic information were considered: in total, 25 stations for NCLDV, prokaryotes, and pico-eukaryotes, and 13 for phages. We ran a Kruskal–Wallis test (‘kruskal.test’ from stats R package [54]) to detect differential OTU abundances between eco-regions, followed by a Benjamini & Hochberg correction to avoid false discovery rate (FDR - *p*.value.bh). Organisms with a *p*-value < 0.05, indicating a difference within groups, were subject to a post-hoc Dunn test (‘dunn.test’ from dunn.test R package [55]) to identify preferential eco-regions for each OTU. From these results, OTUs non-significant Kruskal–Wallis tests (*p*.value.bh >0.05) were assigned to the “ubiquitous” group. In contrast, those with significant *p*-values.bh were classified as EPI, Oxic MES, or OMZ if only the corresponding eco-region was elected according to the Dunn test. Organisms with no significant differences between Oxic MES and OMZ were assigned to Core MES.

### Co-occurrence network inference

For investigation of ecological associations between organisms across eco-regions, a co-occurrence network was inferred. In this analysis, phage samples were not included due to the lower number of stations sampled. Therefore, samples for NCLDV, prokaryotes and pico-eukaryotes from stations 038, 039, 068, 070, 072, 076, 078, 098, 100, 102, 109, 110, 111, 112, 122, 132, 133, 137, 138, 142, 145, 146, 148, 149 and 152 were retained. OTUs with a relative abundance lower than10^−4^ and counting fewer than 5 observations were discarded. Network inferences were performed on CLR transformed data using FlashWeave version 0.18 implemented in Julia version 1.2 [56], using the sensitive and heterogeneous mode. FlashWeave assumes features to be multivariate Gaussian distributed in CLR-transformed space.

We analyzed this global co-occurrence network by delineating communities (or modules) using the Clauset-Newman-Moore algorithm [57]. These modules are subsets of OTUs, obtained by maximizing the co-occurrences within modules and minimizing connections between them. Next, we investigated modules enriched in OTUs from specific eco-regions using Fisher’s exact test using the “fisher.test” function from the stats R package, followed by the Benjamini & Hochberg correction to control the FDR due to multiple testing.

## Results and discussion

**Fig. 2:**
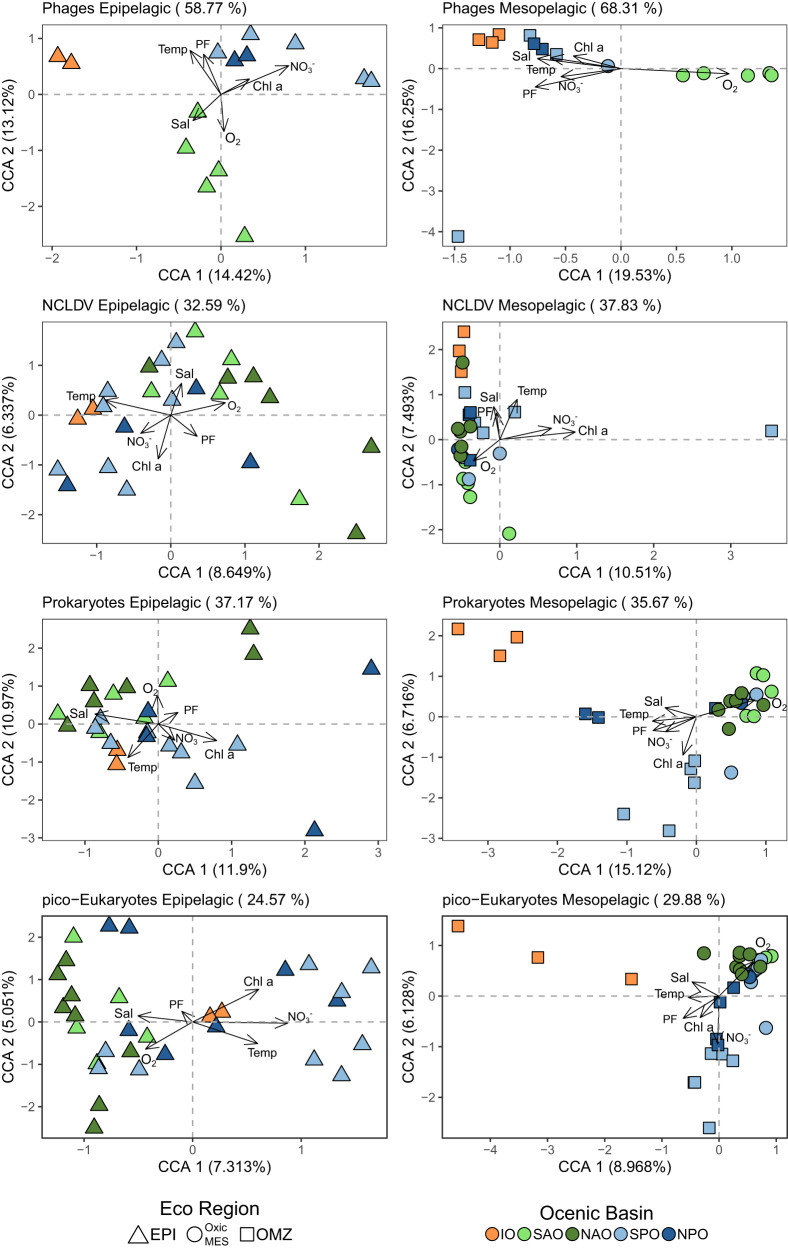
Ordination plot of canonical correspondence analysis (CCA) from epipelagic (left) and mesopelagic (right) communities based on OTU composition. Percentages in parentheses are the amount of variation constrained - in titles represent the total in each analysis, and in the axis represent the correspondent value for each dimension. Arrows represent environmental quantitative explanatory variables with arrowheads indicating their direction of increase. Shapes represent sampling sites. Shape formats represent eco-regions, epi epipelagic, Oxic MES oxic mesopelagic, OMZ oxygen minimum zone mesopelagic. IO Indian Ocean, NAO North Atlantic Ocean, NPO North Pacific Ocean, SAO South Atlantic Ocean, SPO South Pacific Ocean.

Leveraging the resources produced by the *Tara* Oceans project, we deciphered differences between epipelagic and mesopelagic beta-diversity stratification, with a particular emphasis on the role of environmental variables such as temperature, oxygen, salinity, NO_3_^−^, chlorophyll-*a*, and particle flux (see Methods). Here, we combined the diversity information obtained for surface and DCM samples to consider a single epipelagic group. We observed a clear distinction between the mesopelagic and epipelagic communities without loss of signal, as shown in previous studies [6–11], supporting our subsequent analyses reported here (Supplementary Fig. 1, Supplementary Methods Figs. 1 and 2). Consequently, we first investigated differences among physicochemical characteristics of the mesopelagic and epipelagic sampling sites. We observed a high dissimilarity gradient among sites for both layers (Supplementary Fig. 2a, b). Mesopelagic samples were heterogeneously distributed, with most of the points placed distant from the group centroid (located in the center of the cloud of points identified for each group) (Supplementary Fig. 2a). In contrast, epipelagic points displayed a large variance due to the samples positioned apart from the main cluster (Supplementary Fig. 2a). These results underlie the heterogeneity of environmental conditions encountered in both sampled layers, and this environmental variation may be an important factor that can directly influence community composition.

Next, to quantify how much of the differences in the assemblages’ variance can be explained by environmental conditions, we employed canonical correspondence analysis (CCA) using the environmental variables measured at discrete depths as constraint variables. The results showed that abiotic factors explained 40.5% on average of community variance for both layers (Fig. 2). The phage assemblage was the exception, for which about 58% of the epipelagic variation and 68% of the mesopelagic variation could be explained by the variables investigated (Fig. 2). Our analysis also demonstrated a clustering according to the different oceanic basins studied for all the assemblages, confirmed by a PERMANOVA analysis (Fig. 2, Supplementary Table S1). A basin-scale biogeographical structure was already shown for virus, bacteria and protist in the epipelagic layer [58, 59]. Here, we showed that this structuration appears even more pronounced in the mesopelagic samples at global scale.

Furthermore, we assessed the variance of communities regarding each environmental parameter as explanatory variables individually. In contrast with epipelagic communities mainly structured by temperature, as observed elsewhere [6, 8, 11, 12, 60, 61], temperature was only a significant variable structuring the viruses (phage and NCLDV) in the mesopelagic layer. However, oxygen, NO_3_^−^ and particle flux appeared as common environmental drivers governing at least three out of four mesopelagic assemblages (Table 1, complete analysis in Supplementary Table S2).

**Table 1:** ANOVA *p*-value of the variance explained by environmental variables for global model including all tested environmental variables (Global) and for each explanatory variable individually with all the others used as covariables (independently from their order in the model).

Assemblages	Depth	Global	Temp °C	Salinity	O_2_ [µmol/Kg]	NO_3_^−^ [µmol/L]	Chl-a [µmol/m^3^]	Particle flux
Phages	Epi	0.001	0.048	0.132	0.126	0.001	0.308	0.013
Phages	MES	0.001	0.006	0.151	0.001	0.230	0.306	0.001
NCDLV	Epi	0.001	0.001	0.033	0.036	0.083	0.019	0.197
NCDLV	MES	0.002	0.043	0.186	0.005	0.031	0.001	0.048
Prokaryotes	Epi	0.035	0.062	0.088	0.303	0.152	0.106	0.606
Prokaryotes	MES	0.006	0.410	0.590	0.004	0.039	0.575	0.418
pico-Eukaryotes	Epi	0.025	0.108	0.576	0.373	0.065	0.173	0.807
pico-Eukaryotes	MES	0.001	0.168	0.304	0.033	0.001	0.016	0.009

Previous studies have identified oxygen as one of the main drivers of the eukaryotic community structure in OMZ regions [25, 26, 62]. These studies mainly compared community composition along the oxygen gradient within the water column depth, from the surface downwards. However, depth stratification of plankton communities is evident even in regions with high oxygen concentrations, so distinct parameters co-varying with depth must be taken into account in addition to the oxygen gradients [63].

In addition to the physicochemical parameters, our results show that particle flux (derived from UVP measurements) was a significant variable structuring phage, NCLDV and pico-eukaryote mesopelagic assemblages (Table 1, complete analysis in Supplementary Table S2). These observations support previous reports about the high correlation of this environmental factor with phages, finding possible relevance for the carbon pump’s functioning in epipelagic layers [14]. This observation may also reflect the association with virus (phages and NCLDV) inputs from overlaying water layers via sinking particles [64, 65]. Furthermore, Bettarel et al. [66] suggested that marine aggregates can act as virus-factories, where these entities use the adsorbed bacteria to replicate and therefore be massively exported through the water column (one-way motion). The authors also showed that adsorbed bacteria can easily detach from aggregates (two-way motion), which can explain the lack of correlation between prokaryotes and particle flux observed here. On the other hand, pico-eukaryotes were also driven by particle flux. Durkin et al. [67], demonstrated that about 25% of epipelagic diversity can be detected on marine sinking particles and that the particle associated diversity is linked to the size and type of particle (fecal pellet loose or dense, aggregates and detritus).

**Fig. 3:**
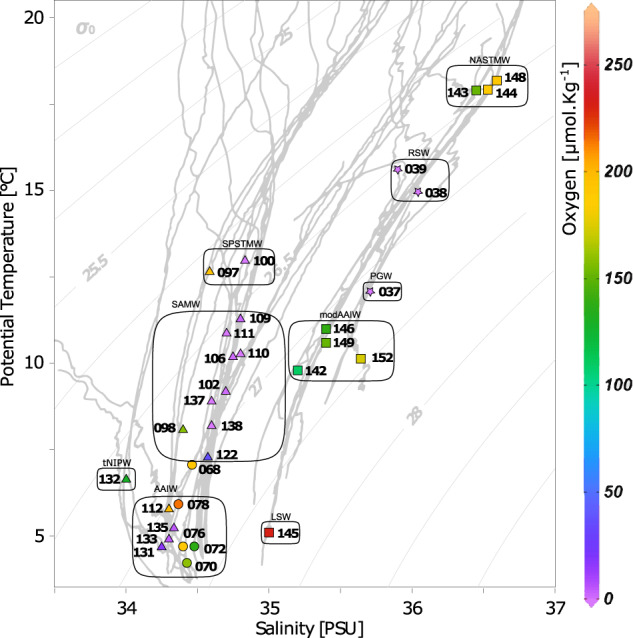
Temperature and salinity plot indicating water mass designation for all mesopelagic samples. Formats represent the different oceanic basins (■ - North Atlantic Ocean, ● - South Atlantic Ocean, ▲- Pacific Ocean, ★- Indian Ocean). Colors indicate the oxygen concentration at the sampling depth. LSW Labrador Sea Water, AAIW Antarctic Intermediate Water, tNPIW transitional North Pacific Intermediate Water, SAMW Subantarctic Mode Water, SPSTMW South Pacific Subtropical Mode Water, modAAIW modified Antarctic Intermediate Water, PGW Persian Gulf Water mass, RSW Red Sea Water mass, NASTMW North Atlantic Subtropical Mode Water.

In situ physico-chemical measurements have revealed the dynamics and fluctuating nature of the ocean, even over a short time scale [68]. The heterogeneity in mesopelagic layers given by deep currents, the impact of surface production, and the low mixing levels may favor a diversification in the mesopelagic community living in different water masses, leading to species adaptation-acclimation. The *Tara* Oceans expedition route included samples from common or distinct water masses defined by temperature/salinity profiles - T/S, comprising regionally connected or unconnected stations. We identified nine different water masses in the mesopelagic sampled locations (Fig. 3). We could confirm significant differences among mesopelagic communities sampled in these different water masses based on the PERMANOVA test (Table 2). This result indicates that the oceanic patchiness created by distinct water masses significantly shape community beta-diversity in the mesopelagic layer, which would imply it to be a critical component for mesopelagic community variation for all the assemblages studied (phages, NCLDV, prokaryotes, and pico-eukaryotes). Thus, we hypothesize that this result may be explained by two non-exclusive causes related to water masses: (i) past common origin among water masses that have drifted or (ii) constant connectivity by ocean circulation between sampled sites belonging to the same water mass.

**Table 2:** Proportion of the assemblages variation explained by water masses using the Permutation multivariate analysis of variance (PERMANOVA)

Assemblage	DF	Sum of squares	Mean squares	F	R^2^	Pr
Phages	4	1513.1	378.27	1.36	0.37	0.1
NCDLV	8	3136.6	392.08	1.72	0.45	0.01
Prokaryotes	8	13584	1698.02	2.37	0.53	0.001
pico-Eukaryotes	8	71998	8999.8	1.54	0.35	0.001

We addressed another lingering question, resolving planktonic community signatures of Oxic MES and OMZ regions from those observed in epipelagic layer. For this, we classified OTUs into three eco-regions: 1) EPI, 2) Oxic MES, and 3) OMZ. OTUs were classified as Core MES when commonly present in Oxic MES and OMZ samples. Taxa that were either equally abundant in all three eco-regions or not statistically confirmed to a single eco-region were classified as ubiquitous (Supplementary Fig. 3, Supplementary Tables S3–S7). Using this approach, we could identify ubiquitous taxa that are likely to thrive in a wide range of environmental conditions, or that may be detected in mesopelagic samples due to the simple vertical movement of sinking particles. This classification should help avoid putative biases inherent to the metabarcoding methodology.

**Fig. 4:**
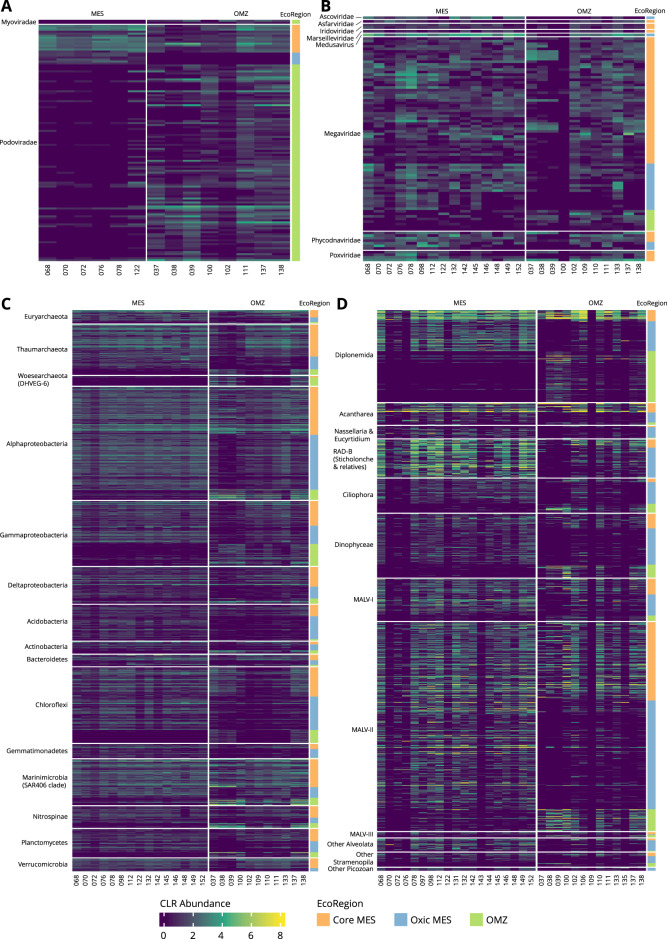
Heatmaps occurrence of OTUs assigned to mesopelagic eco-regions. **A** Phages, **B** NCLDV, **C** Prokaryotes and **D** pico-Eukaryotes.

More specifically, we were able to identify Oxic MES and OMZ signatures mainly at the infra-taxonomic level (OTU-species) for all biotic groups investigated (Fig. 4, Supplementary Figs. 3–7, Supplementary Tables S3–S7). This reflected the wide ecological niche occupied by the different species at a higher taxonomic level (i.e. family). At the species level, we observed large taxonomic plasticity of OTUs that occurred equally in both Oxic MES and OMZ samples, called Core MES. However, most OTUs are not yet classified at the infra-taxonomic level (Supplementary Tables S3–S7). This observation reflects the knowledge gap about the biodiversity and functional plasticity of species thriving in this ecosystem.

The great majority of phage taxa (93.51%) occurred at similar abundance in all eco-regions (ubiquitous). Surprisingly, we did not identify any OTUs assigned specifically to an EPI eco-region, meaning that almost all taxa observed in the epipelagic layer were also present in the mesopelagic layer (Supplementary Figs. 3, 4). This observation supports the seed-bank hypothesis raised by Brum et al. [60], and the correlation to the sinking particles observed here. On the other hand, we detected taxa specific to the mesopelagic layer, mostly related to the OMZ eco-region (Fig. 4a, Supplementary Figs. 3–4). This mesopelagic specificity agrees with the sharp increase in marine phage micro-diversity following depth, as previously shown by Gregory et al. [6]. Our results emphasize that one cause for phage stratification in the water column might be the adaptation to the mesopelagic environment. Two hypotheses arise here, 1) the environment acts as a strong driver, directly selecting phages independently of their hosts, and 2) there is higher phage-host specificity in the mesopelagic layer, promoting phage selection. Following the first hypothesis, we can posit that the environment can directly impact phage assemblage composition. The direct contact with the environment of free phage entities (released from their hosts) may reduce infectivity, degrade, or remove virus particles, and adversely affect adsorption to the host [69]. This direct environmental effect over marine phages was reported for different ionic gradients [70], daylight conditions, and temperature [71]. However, the enrichment of prokaryotic OTUs specific to mesopelagic regions (Fig. 4, Supplementary Figs. 3, 6), especially in OMZs, does not exclude the phage-host indirect selection relationship.

We found 136 mesopelagic-specific NCLDV OTUs out of 5538 in both oxic and OMZ eco-regions. Even though it represents a small number, these OTUs were highly abundant (Fig. 4b, Supplementary Figs. 3, 5). Most of the mesopelagic-specific NCLDV OTUs corresponded to the Core MES group (OMZ = 18 OTUs, Oxic MES = 31 OTUs, Core MES = 87 OTUs- Supplementary Table S3). NCLDV can encode genes such as transporters for ammonium, magnesium, and phosphate that are important in marine oligotrophic areas [72]. This characteristic can improve the host’s fitness in the short-term and ultimately favor NCLDV fecundity and endurance. This property is named NCLDV-mediated host reprogramming [72]. Our results therefore indicate that these entities are less diverse in mesopelagic waters and may successfully infect a wide range of hosts adapted to different oxygen concentrations.

Among the planktonic microorganisms, prokaryotes have been, so far, the most investigated group in OMZ regions, especially in the Pacific Ocean [20, 21]. We could better distinguish the prokaryotic mesopelagic signatures between Oxic MES and OMZ, confirming the influence of oxygen reported here and in previous studies [20–22] (Fig. 4c, Supplementary Figs. 3, 6). We observed similar occurrences and abundances for the OMZ signature taxa in the Indian Ocean stations (IO - 037, 038, 039) and in stations PO - 100, 137, and 138 from the Pacific Ocean (Fig. 4c). These Pacific stations are located in the open ocean (PO - 137 and 138 located in the Equatorial upwelling zone and station PO - 100 in the South Pacific Subtropical Gyre). They present a strong upwelling signature, disclosing an intense decrease in oxygen concentration almost reaching shallow waters. Likewise, the sampling stations in the Indian Ocean are located in well-stratified waters, markedly characterized by the abrupt decrease of oxygen concentration below the thermohaline at 100–120 m depth, especially for stations 038 and 039. At the mesopelagic layer of the Indian Ocean stations, the oxygen concentration ranges from 0.83 to 3 μmol/kg, characterizing functionally anoxic waters since aerobic metabolisms cannot be sustained at this oxygen level [73]. The other OMZ stations in the Pacific Ocean (PO - 102, 109, 110, 111) are located in coastal areas. Although they are also under the influence of upwellings, with low oxygen content, the oxygen level does not correspond to anoxic conditions, so they are classified as suboxic waters. This microoxic condition of this environment is sufficient to completely alter the microbial metabolism delineating the community composition in these sites. In addition, differences in the formation of offshore and coastal upwelling, for instance, or the influence of river runoffs, transporting anthropogenic nutrient enrichment from the continent to coastal areas [73], could be crucial in supporting the differences we observed in OMZ communities.

The same clear enrichment in both OMZ anoxic and suboxic samples was observed for the pico-eukaryotic groups Diplonemida, MALV-II, and Dinophyceae, suggesting these OTUs as the true OMZ eukaryotic signatures (Fig. 4d). Some OTUs of these groups exhibited similar occurrences in the anoxic Indian and Pacific Oceans but not in suboxic samples from the Pacific Ocean. However, we observed a lower number of pico-eukaryotic taxa in the OMZ eco-region, the prevailing OTUs being specific to Oxic MES locations in most cases.

**Fig. 5:**
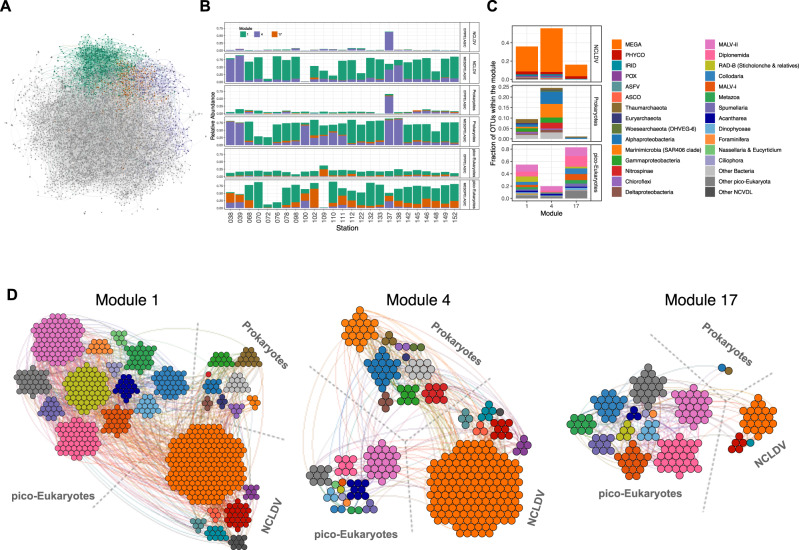
Co-occurrence network in epipelagic and mesopelagic communities. **A** Global network, with connected modules for OMZ (purple and orange) and Oxic MES (green) highlighted. **B** Relative taxa abundance in each module in each station and depth. **C** Relative number of OTUs classified in taxonomic groups. **D** Network representation of modules.

Another step to better understand mesopelagic community dynamics is to dissect the ecological relationships among species that thrive in this layer. Co-occurrence networks can indicate how the environment may structure the community acting as a filter for resident species [74]. They can also give us glimpses of organisms’ potential ecological interactions based on species connectivity [74, 75]. Combining the NCLDV, prokaryote, and pico-eukaryote data, we inferred a network containing 6154 nodes and 12,935 edges (Fig. 5a, Table 3). Due to the lower number of stations sampled for phages, we excluded this group from the analysis. We found mainly positive relationships (94%), suggesting a predominance of putative biotic interactions (e.g. competition, symbiosis) rather than taxa avoidance or exclusion. This dominance of positive relations was also reported for epipelagic plankton communities [36, 76]. The global network had a modularity value greater than 0.4 (Table 3), indicating that the network has a modular structure [73]. Applying a community detection algorithm [57] on this global graph, we were able to delineate 36 distinct modules (or subnetworks), presumably corresponding to ecological communities. Three of them were mainly composed of OTUs significantly enriched in mesopelagic OTUs (Oxic MES enriched module 1, *p*-value.bh = 1.29e^−153^ and OMZ enriched modules 4, *p*-value.bh = 1.90e^−51^, and 17, *p*-value.bh = 2.1e^−13^; Fig. 5). Together, these three modules covered almost the total richness found in the mesopelagic zone (Fig. 5b), and presented similar values for the average degree, clustering coefficient, and average path length (Table 3). These parameters indicate a network complexity [74], hinting at distinct ecological niches within the mesopelagic layer.

**Table 3:** Network topological features derived from global analysis including NCDLV, prokaryotes and pico-Eukaryotes samples in epipelagic and mesopelagic depths.

Parameter	Global	Mod 1	Mod 4	Mod 17
Nodes	6154	731	323	175
Positive edges	12193	1236	480	223
Negative edges	742	70	49	9
Av. Degree	4.20	3.57	3.28	2.65
Clustering	0.03	0.03	0.09	0.05
Density	0.00	0.00	0.01	0.02
Average path length	7.28	6.01	6.27	6.30
Betweenness	0.01	0.05	0.10	0.22
Degree Centralization	0.00	0.01	0.02	0.04
Modularity	0.47	0.60	0.67	0.66

More precisely, the OMZ modules were composed of a few connected nodes (323 and 175 nodes for modules 4 and 17, respectively - Table 3), potentially indicating two distinct OMZ community niches. The Oxic MES module 1 counted more nodes composing the network associations (731 nodes), and all modules presented a variation in taxonomic composition and proportions. OMZ module 4 (OMZ-4) contained mainly prokaryotic (23%) and NCLDV (55%) OTUs (Fig. 5c, d). Among the prokaryotes, we detected taxa previously determined as representing OMZ signatures (Nitrospinae, Marinimicrobia SAR 406, and Planctomycetes). As this module contains both prokaryotes and NCLDV OTUs, it suggests the existence of a confounding factor not captured in the dataset (such as large eukaryotes), as NCLDV are known to be specific to eukaryotes. Nevertheless, this correlation has been observed independently in another study [12]. On the other hand, module 17 (OMZ-17) is mainly composed of pico-eukaryotes OTUs (83%) (notably MALV-II (14%) and Diplonemida (17%) previously indicated as OMZ signatures) and NCLDV (16%) as expected due to virus-host relationships. Module 1 is taxonomically more diverse but consisted mainly of NCLDV and pico-eukaryotes. These groups accounted for 36 and 55%, respectively, of OTUs in this module (Fig. 5c, d). NCLDV contributed to 598 associations (edges) in mesopelagic module 1, of which 177 occurred between NCLDV and pico-eukaryotes. NCLDV from the *Mimiviridae* family are the most numerous taxa in all three mesopelagic modules. *Mimiviridae* is a very abundant family in the ocean, present in various size ranges from piconanoplankton (0.8–5 μm) up to mesoplankton (180–2000 μm) [9, 75]. This observation supports our finding that NCLDV are a prosperous group in mesopelagic waters, undertaking different strategies to endure in such environmental conditions. In all three modules, we observed the presence of Foraminifera, of which some species can use nitrate over oxygen as an electron acceptor, favoring their survival in OMZ regions [77].

Our converging results suggest that the mesopelagic zone can be characterized by at least three well-defined ecological niches (Oxic MES, OMZ-4 and OMZ-17), with established conditions and resources (abiotic and biotic) that allow the survival of a specific communities in these environments. Observed differences between OMZ and Oxic MES networks suggest a potential loss of connections and interactions among mesopelagic community members, directly affecting ecosystem stability due to habitat change.

## Conclusions

In this study, we explored mesopelagic pico-plankton ecological structuring and concluded that this component of oceanic plankton is heterogeneous with respect to environmental conditions. We could pinpoint the relevance of oxygen for all assemblages and the relation of particle flux with phages, NCLDV and pico-eukaryotes. These results reinforce the need to better understand the mesopelagic ecosystem in order to improve our comprehension of carbon export through the biological carbon pump in the twilight zone. Also, we show that intermediated water masses defined by their T/S profiles can explain the differences in the observed mesopelagic pico-plankton structure, pointing to the role of a set of environmental parameters for community composition.

By establishing eco-regions (Epipelagic, Oxic MES, and OMZ), we were able to discriminate specific mesopelagic signatures OTUs across all Life’s domains. While we recovered known markers for Oxic MES and OMZ regions at high taxonomic levels, we also found that most of these OTU signatures are observed at low taxonomic levels, which sometimes cannot be resolved using existing databases. Combining these OTU profiles within co-occurrence networks, we proposed three niches with biotic and abiotic conditions that appear to characterize mesopelagic ecosystems.

Limited access to data is usually the bottleneck for knowledge of mesopelagic dynamics. Our study benefits from a larger number of organism samples and distinct oceanic provinces. This allowed us to integrate these data and thus obtain an expanded vision of mesopelagic community structure and dynamics. Our results emphasize the need for a better understanding of mesopelagic life, particularly by improving our knowledge of oxic and oxygen-low mesopelagic-dwelling communities. This effort is especially necessary as climate change can be expected to expand marine OMZs in the future.

## Supplementary Information

Supplementary Material and Methods

Supplementary Figure S1

Supplementary Figure S2

Supplementary Figure S3

Supplementary Figure S4

Supplementary Figure S5

Supplementary Figure S6

Supplementary Figure S7

Supplementary Tables

## Data Availability

All data analyzed during this study are included in this published article in the Supplementary Information file.
