# Supplementary material for: Ocean-wide comparisons of mesopelagic planktonic community structures
Source: ISME Commun. 2023 Aug 18;3:83. doi: 10.1038/s43705-023-00279-9 (PMC10439195; doi:10.1038/s43705-023-00279-9)
Supplement: Supplementary file 1 — Supplementary Material and Methods [file 43705_2023_279_MOESM1_ESM.docx]

Supplementary Material and Methods

The present document details information about the *Tara* Oceans data availability and the pre-processing used for the analyses reported in Rigonato et al. 2021 “**Ocean-wide comparisons of mesopelagic planktonic community structures**”. Note that here we standardize the term “OTU” as “operational taxonomic unit” *sensu latu*, independent of clustering technique since taxonomy for the different assemblages was obtained using different methodologies. Also, it should be noted that keeping the original OTU definition datasets allowed direct comparison with previous work within the TARA Consortium.

# Data Processing

## Phage populations

Viral-phage contigs were assembled and identified from the 0.22 µm-filtered seawater viromes from the *Tara* Oceans expedition 2009-2013 and Malaspina 2010 global circumnavigation expedition using the methods described in (Gregory et al. *2019*). Prodigal 2.6.1 (Hyatt et al. 2010) was used to call ORFs (genes and proteins) using the ‘meta’ setting. The resulting proteins were clustered based on blast-defined sequence similarities and granularity of ‘2’ with MCL (Enright et al. 2002). The resulting genes were searched for four different primer sets for *gp23* (Table S2), the major capsid protein found in T4-related members of *Myoviridae*, and 10 different primer sets for *polA* (Table S2), the DNA polymerase found in *Podoviridae* (Adriaenssens et al. 2014). Because the genes are not always complete in metagenomic datasets, exact primer hits (100% nucleotide identity) of only the forward or the reverse primer were considered hits. Genes that hit these primers were translated into proteins and the MCL-defined protein clusters (PCs) that contained >=1 hit were extracted. PCs were annotated by running a combination of the reciprocal best blast hit analyses against the KEGG database (Kanehisa 2002) and blast against the UniProt Reference Clusters database (Suzek et al. 2007), searching for matches against the InterPro protein signature database using InterProScan (Zdobnov et al. 2001), and run hmmsearch against Pfam entries (Bateman et al. 2004) and then manually curated for *gp23* and *polA* genes, respectively. The *gp23* and *polA* proteins were matched to their gene sequences and dereplicated at 95% nucleotide identity across 100% the length into clusters using CD-HIT-EST (Li and Godzik 2006). The abundances of the *gp23* and *polA* clusters were calculated by pooling the abundances of the viral contigs containing the *gp23* or *polA* genes, respectively, per station.

In total, 848,507 viral contigs were identified. Viral contigs were grouped into populations if they shared ≥ 95% nucleotide identity across ≥ 80% of the genome (sensu) (Brum et al., 2015) using nucmer (Kurtz et al., 2004). With this, 488,130 total viral populations, here called OTUs, were obtained by the authors. For the present work, we used only *pol*B and *gp23* populations (9,048 OTUs).

Table S2. The *gp23* and *polA* primer sets used to search viral gene sequences

| **Signature Gene** | **Primers (5’-3’)** |
| --- | --- |
| *gp23* | MZIA1: TGT TATIGGTATGGTICGICGTGCTAT  CAP8: TGAAGTTACCTTCACCACGACCGG    MZIA1bis: GATATTTGIGGIGTTCAGCCIATGA  MZIA6: CGCGGTTGATTTCCAGCATGATTTC    T4superF1: [TET]GAYHTIKSIGGIGTICARCCIATG  T4superR1: [6FAM]GCIYKIARRTCYTGIGCIARYTC    G23-For: ACWGGWCTKATYTTCGCAATG  G23-Rev: AYTTYTCAACWGACCADCKACC |
| *polA* | T7DPol230F: ARGARMRIAAYGGIT  T7DPol510R: GTRTGDATRTCICC    HECTORPol19F: GCAAGCAACTTTACTGTGG  HECTORPol711R: CGAGAGATACACCAACGAA    HECTORPol563F: CTTCTCAGTTTTCTGTT  HECTORPol800R: GCAAGCAACTTTACTGT    PARISPol25F: ATACTACACGCTACTCTGG  PARISPol701R: GAGTGGCAAGAGGAGTTAT    PARISPol480F: AAGTTGTGCTTCTGGTA  PARISPol786R: ATACTACACGCTACTCT    Podo-F: GACACHCTYRTVHTGTCWMGWYTG  Podo-R2: MCKACCRTCYARDCCYTTMAK    CP-DNAP-349F: CCAAAYCTYGCMCARGT  CP-DNAP-533Ra: CTCGTCRTGSACRAASGC  CP-DNAP-533Rb: CTCGTCRTGDATRAASGC    DPOL-341Fd: CCNAAYYTNGSNCARGTNCC  DPOL-534Rd: TGNWRYTCRTCRTGNAYRAA    DPOL-349Fd: CCNAAYYTNGSNCARGT  DPOL533Rd: TCRTCRTGNAYRAANGC |

##

## Nucleo-Cytoplasmic Large DNA Virus - NCLDV

NCLDV sequence data was obtained from Endo et al., 2020. First, to assess the community composition of NCLDVs, *polB* was used as a marker gene for NCLDVs. 29,315 PolB sequences were obtained from the OM-RGC.v2 by using an in-house profile hidden Markov model of NCLDV *polB* sequences using the software HMMER, hmmsearch (version 3.1) with a threshold e-value of < 1 × 10^–5^.

To filter NCLDV derived sequences from those of other domains of life a phylogenetic tree was built based on 211 PolB reference protein sequences (eukaryotes, bacteria, archaea, phages, and NCLDVs). This tree included sequences from eight proposed families of NCLDVs: *Mimiviridae* (synonymous with *Megaviridae*), *Phycodnaviridae*, *Pithoviridae*, *Marseilleviridae*, *Ascoviridae*, *Iridoviridae*, *Asfarviridae*, and *Poxviridae*, and a sequence from *Medusavirus*, a novel NCLDV clade. The MAFFT-linsi (Kato and Standley 2013) was used for alignment and RAxML for maximum likelihood tree inference (Stamatakis 2006). NCLDV sequences derived from OM-RGC.v2 were aligned against the reference alignment using the MAFFT ‘addfragments’ option and then placed in the tree using the pplacer software (Matsen et al. 2010).

Next, NCLDV genes from the OM-RGC.v2 were used to obtain abundance profiles. Only samples from the pico- (0.22–1.6 μm or 0.22–3.0 μm) and femto- (<0.22 μm) size fractions were included. The sum of length-normalized *pol*B abundances ranged from 5.3 to 22,847.5 across samples, so the samples for which the sum of length-normalized *pol*B abundance was less than 50 (set as a proxy for low NCLDV frequency) were removed from the analysis. The abundance matrix was then standardized by the sample with the lowest sum of length-normalized *polB* abundance values.

## 16S rRNA gene mitags

The 16S rRNA gene dataset used in this manuscript was derived from environmental metagenomes following the approach of Logares et al. 2014, named “mitags”, and thoroughly described in Sunagawa et al. 2015 and Salazar et al. 2019. This approach is considered a powerful alternative to 16S rRNA gene amplicons as it overcomes PCR biases related to amplification and primer mismatch (Logares et al., 2014).

Briefly, 16S rRNA gene reference sequences from the SILVA database (Quast et al., 2013) were clustered at 97% sequence identity to balance the unequal taxa representation and define OTUs at the genus level. Then, the recruited mitags were mapped to cluster centroids of taxonomically annotated SILVA 16S reference sequences using USEARCH v9.2.64 (Edgar, 2010). Only the mitags mapping to a unique reference sequence were used to compute abundances at the OTU level. Mitags that mapped to more than one reference (OTU) were processed at a higher taxonomic level (domain, phylum, class, order, family or genus) common to all mapped OTUs. The abundance OTU table was generated by counting the number of mitags assigned to each taxon in each sample and the number of unassigned mitags. In total, 23,986 mitags were identified.

## 18S rRNA gene Metabarcoding

For 18S rRNA gene OTUs, conventional PCR marker amplification was used to investigate the eukaryotic diversity in Tara Ocean samples from de Vargas et al. 2015 and Ibarbalz et al. 2019. To obtain these OTUs, the authors applied a fast clustering method called *swarm* (Mahé et al., 2015), which can deliver high-resolution clusters, down to single-nucleotide differences with some additional post-processing, satisfying the ASV definition of Callahan et al. 2017. The *swarm* approach is free of arbitrary global clustering thresholds and input-order dependency that are the two main fundamental problems observed in traditional methods for clustering OTUs (Mahé et al., 2015).

The number of sequencing reads for each OTU was used as a proxy for abundance. OTUs were annotated using an in-house version of the PR2 database (Guillou et al. 2012, del Campo et al. 2018). In total, 474,303 OTUs were obtained.

A detailed explanation of read processing is available at <http://taraoceans.sb-roscoff.fr/EukDiv/>.

# Epipelagic Data Merging

Given the focus of the present study, we reclassified the samples as “Epipelagic” (SRF + DCM) and “Mesopelagic” (MES).

Here, we present results that support this decision, showing that SRF and DCM deviations are minor compared to MES samples. To combine both SRF and DCM samples, reads from the same OTUs at the same station were summed and then normalized by the total number of reads.

Figure 1 shows that the beta-diversity of the merged samples (EPI mod) do not differ from those obtained initially (*orig* SRF and DCM separately). The similarity between EPI and SRF or DCM was statistically confirmed by performing the ANOVA-like hypothesis test ANOSIM (Table S3; Clarke 1993). ANOSIM ranks the dissimilarity matrix to values between 0 and 1 (R values close to 0 suggest an even distribution and R values close to 1 suggest dissimilarity between groups) to compare the mean of ranked dissimilarities between groups to the mean of ranked dissimilarities within groups.

Figure 2 shows the dispersion of measures of the environmental samples recovered in situ during the biological sampling. We can observe that SFR and DCM are not statistically different.


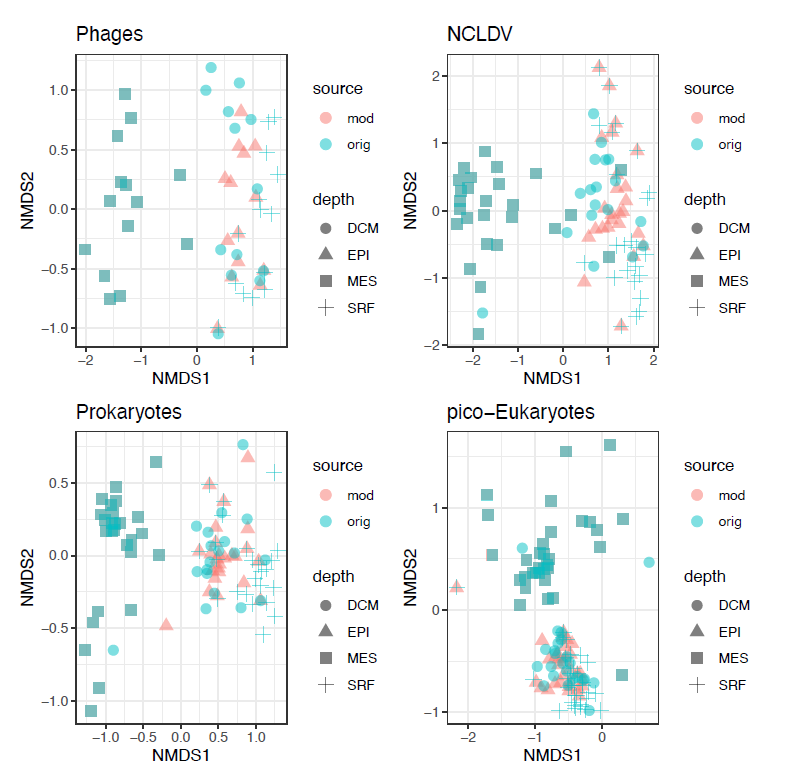


Figure 1. NMDS plots using the original datasets (*orig*, containing SRF, DCM, and MES layers) and the modified (*mod,* containing EPI and MES) datasets. MES samples are equal in both *mod* and *orig* datasets. Stress: 0.17 (virus), 0.14 (girus), 0.086 (Prokaryotes), 0.17 (Eukaryotes).


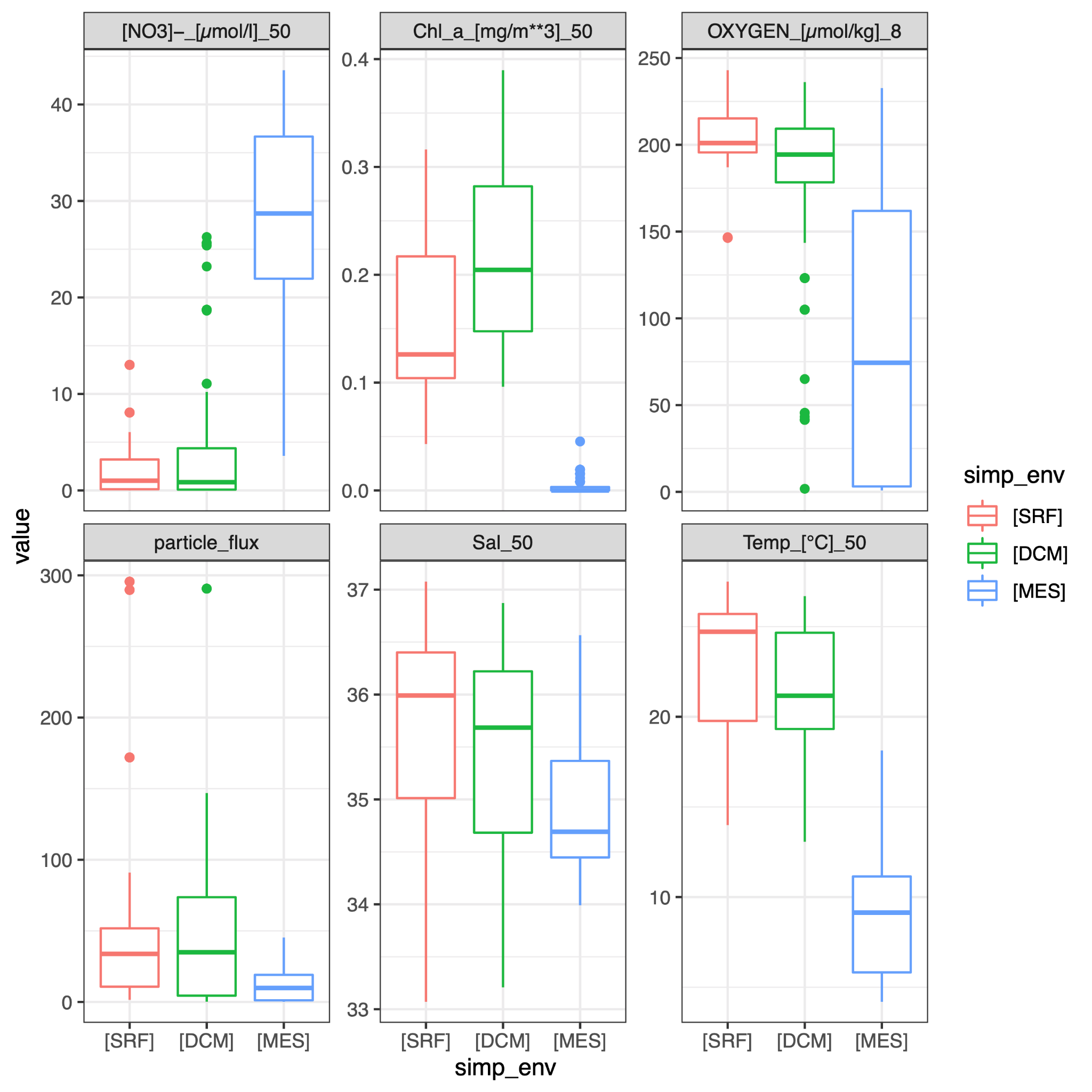
Figure 2. Environmental Parameters of Samples. An outlier in the particle flux (~3428) was removed from the plot. Y-axis is in original parameter units from PANGEA (<https://doi.pangaea.de/10.1594/PANGAEA.875582>).

Table S3 ANOSIM statistics supporting the SRF and DCM data merging into EPI

| Assemblage | Depth1 | Depth2 | R | adj.p.val |
| --- | --- | --- | --- | --- |
| Phages | EPI | MES | 0,901 | 0,001 |
| Phages | EPI | DCM | 0,024 | 0,257 |
| Phages | EPI | SRF | 0,075 | 0,084 |
| Phages | MES | DCM | 0,883 | 0,001 |
| Phages | MES | SRF | 0,877 | 0,001 |
| Phages | DCM | SRF | 0,209 | 0,004 |
|  |  |  |  |  |
| NCLDV | EPI | MES | 0,772 | 0,001 |
| NCLDV | EPI | DCM | 0,045 | 0,118 |
| NCLDV | EPI | SRF | 0,028 | 0,136 |
| NCLDV | MES | DCM | 0,716 | 0,001 |
| NCLDV | MES | SRF | 0,815 | 0,001 |
| NCLDV | DCM | SRF | 0,096 | 0,020 |
|  |  |  |  |  |
| Prokaryotes | EPI | MES | 0,933 | 0,001 |
| Prokaryotes | EPI | DCM | 0,087 | 0,025 |
| Prokaryotes | EPI | SRF | 0,127 | 0,006 |
| Prokaryotes | MES | DCM | 0,855 | 0,001 |
| Prokaryotes | MES | SRF | 0,953 | 0,001 |
| Prokaryotes | DCM | SRF | 0,172 | 0,001 |
|  |  |  |  |  |
| pico-Eukaryotes | EPI | MES | 0,731 | 0,001 |
| pico-Eukaryotes | EPI | DCM | 0,140 | 0,002 |
| pico-Eukaryotes | EPI | SRF | 0,038 | 0,038 |
| pico-Eukaryotes | MES | DCM | 0,525 | 0,001 |
| pico-Eukaryotes | MES | SRF | 0,773 | 0,001 |
| pico-Eukaryotes | DCM | SRF | 0,179 | 0,001 |

# References:

Adriaenssens, EM, and Cowan, DA. Using signature genes as tools to assess environmental viral ecology and diversity. *AEM*, AEM-00878, 2013.

Bateman, A et al. "The Pfam protein families database." *Nucleic acids research* 32.suppl_1 D138-D141, 2004:.

Brum JR, et al. Patterns and ecological drivers of ocean viral communities. *Science* 348: 1261498–1261498, 2015.

Callahan, BJ, McMurdie, PJ, and Holmes, SP. Exact sequence variants should replace operational taxonomic units in marker-gene data analysis. *The ISME journal* 2639-2643, 2017.

Clarke, KR. Non-parametric multivariate analyses of changes in community structure. *Aust J Ecol* 18**:**117-43, 1993.

de Vargas C, et al. Eukaryotic plankton diversity in the sunlit ocean. *Science* 348: 1261605–1261605, 2015.

Del Campo, Javier, et al. EukRef: phylogenetic curation of ribosomal RNA to enhance understanding of eukaryotic diversity and distribution. *PLoS biology* 16.9 e2005849, 2018.

Edgar, Robert. *Usearch*. Lawrence Berkeley National Lab. (LBNL), Berkeley, CA (United States), 2010.

Endo H, Blanc-Mathieu R, Li Y, Salazar G, Henry N, Labadie K, et al. Biogeography of marine giant Phageses reveals their interplay with eukaryotes and ecological functions. *Nat Ecol Evol* 4: 1639–1649, 2020.

Enright, AJ, Van Dongen, S, Ouzounis, CA. An efficient algorithm for large-scale detection of protein families. *Nucleic acids research* 30.7 1575-1584, 2002.

Gregory AC, Zayed AA, et al. Marine DNA Viral Macro- and Microdiversity from Pole to Pole. *Cell* 177: 1109-1123.e14, 2019.

Guillou, L, et al. The Protist Ribosomal Reference database (PR2): a catalog of unicellular eukaryote small sub-unit rRNA sequences with curated taxonomy. *Nucleic acids research* 41.D1 D597-D604, 2012.

Hyatt, D, et al. Prodigal: prokaryotic gene recognition and translation initiation site identification. *BMC bioinformatics* 11.1, 119, 2010.

Ibarbalz FM, et al. Global Trends in Marine Plankton Diversity across Kingdoms of Life. *Cell* 179: 1084-1097.e21, 2019.

Kanehisa, M, et al. The KEGG databases at GenomeNet. *Nucleic acids research* 30.1, 42-46, 2002.

Katoh, K, and Standley, DM. MAFFT multiple sequence alignment software version 7: improvements in performance and usability. *Mol. Biol. Evol.* 30, 772–780, 2013.

Kurtz, S, et al. Versatile and open software for comparing large genomes. *Genome biology* 5.2, 1-9, 2004.

Li, W., and Godzik, A. Cd-hit: a fast program for clustering and comparing large sets of protein or nucleotide sequences. *Bioinformatics* 22.13, 1658-1659, 2006.

Logares R, et al. Metagenomic 16S rDNA Illumina tags are a powerful alternative to amplicon sequencing to explore diversity and structure of microbial communities. Environ Microbiol. Sep;16(9):2659-71, 2014.

Mahé, F, et al. Swarm v2: highly-scalable and high-resolution amplicon clustering. *PeerJ* 3 e1420, 2015.

Matsen, FA, Kodner, RB, Armbrust, EV. pplacer: linear time maximum-likelihood and Bayesian phylogenetic placement of sequences onto a fixed reference tree. *BMC Bioinform.* 11, 538, 2010.

Quast, C., *et al*. The SILVA ribosomal RNA gene database project: improved data processing and web- based tools. *Nucleic Acids Res* **41:** D590–D596, 2013.

Stamatakis, A. RAxML-VI-HPC: maximum likelihood-based phylogenetic analyses with thousands of taxa and mixed models. *Bioinformatics* 22, 2688–2690, 2006.

Suzek, BE, et al. UniRef: comprehensive and non-redundant UniProt reference clusters. *Bioinformatics* 23.10, 1282-1288, 2007.

Zdobnov, EM, and Apweiler, R. InterProScan–an integration platform for the signature-recognition methods in InterPro. *Bioinformatics* 17.9, 847-848, 2001.
