# Supplementary figures and images for: Ocean-wide comparisons of mesopelagic planktonic community structures
Source: ISME Commun. 2023 Aug 18;3:83. doi: 10.1038/s43705-023-00279-9 (PMC10439195; doi:10.1038/s43705-023-00279-9)

Phage (Stress = 0.1 )

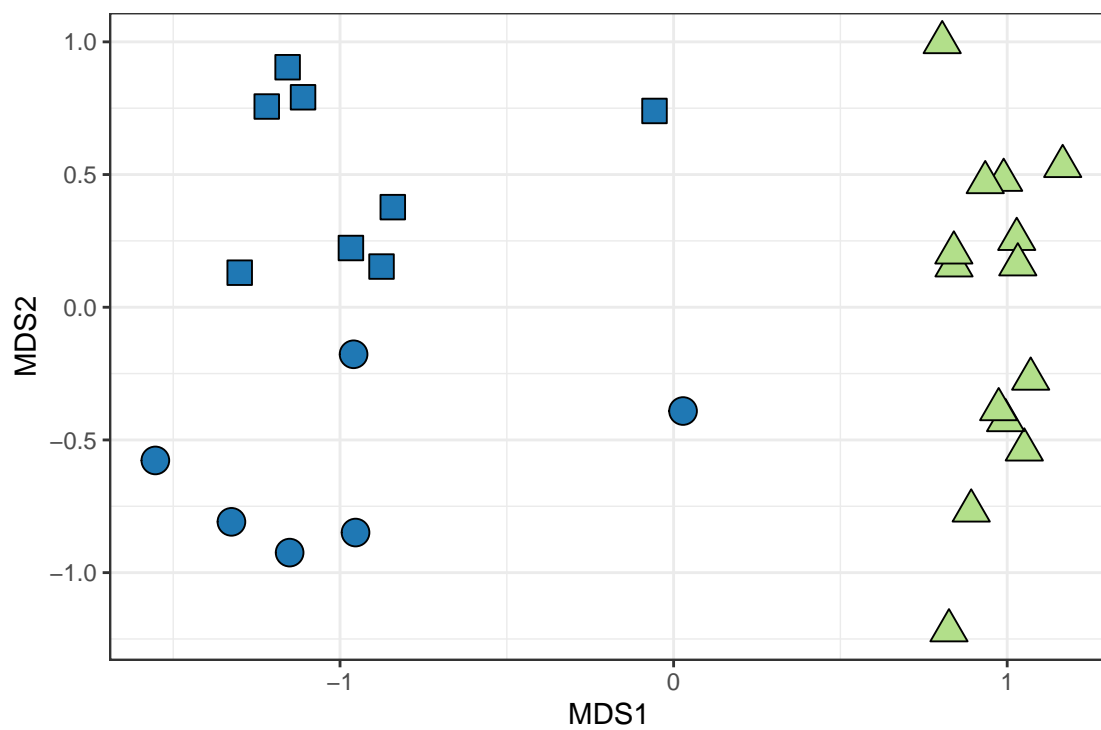

NCLDV (Stress = 0.09 )

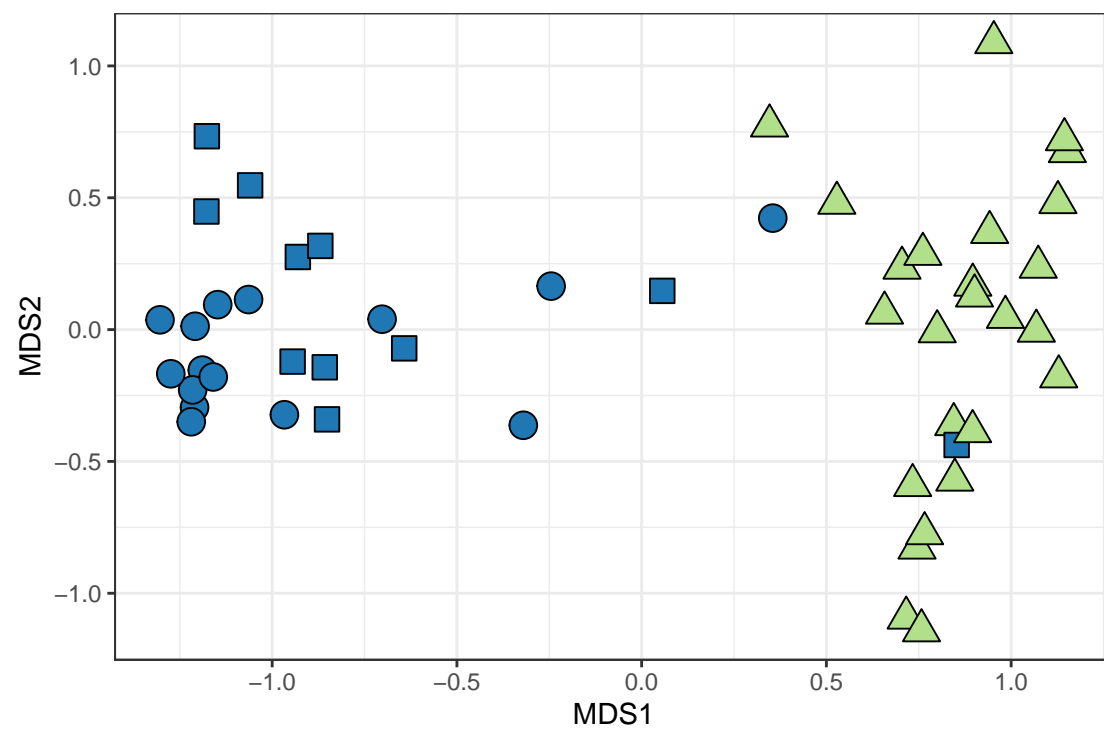

Prokaryote (Stress = 0.06 )

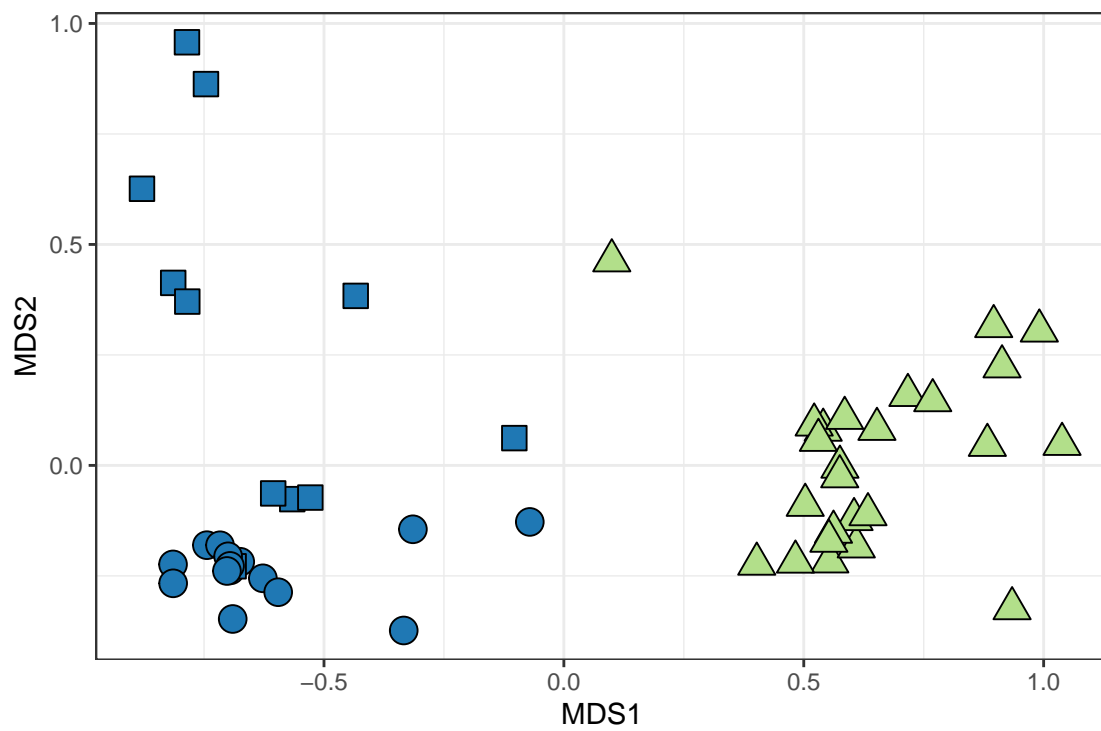

Eukaryote (Stress = 0.08 )

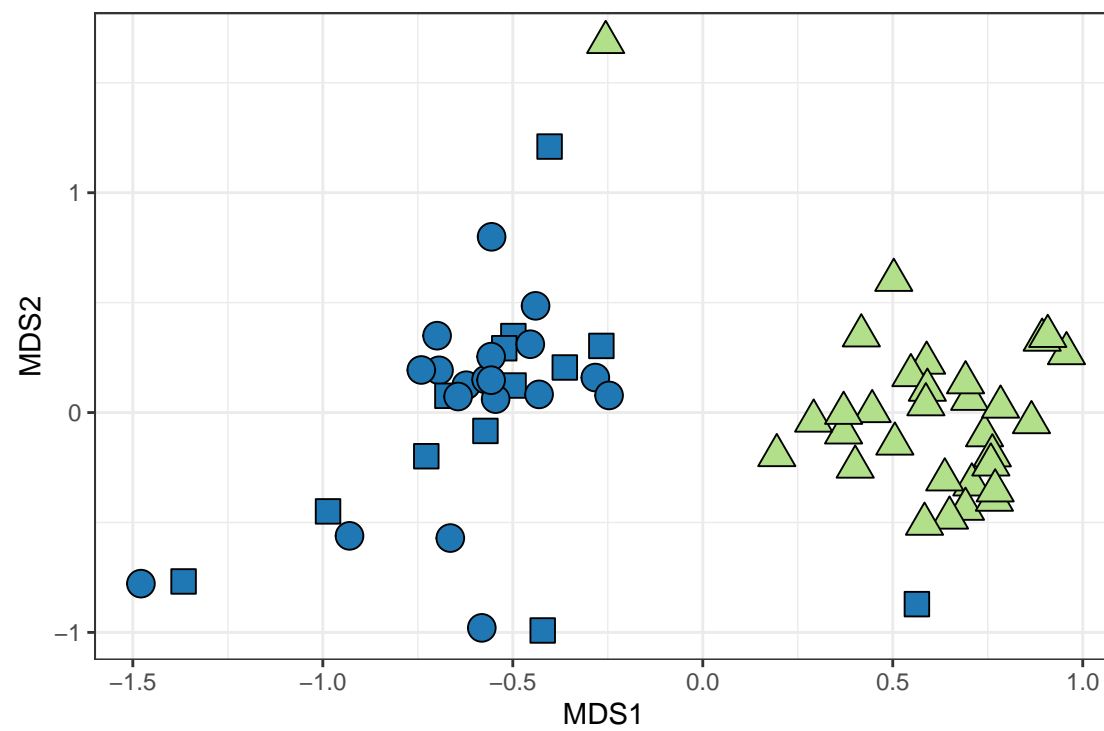

Supplement: Supplementary file 2 — Supplementary Figure S1 [file 43705_2023_279_MOESM2_ESM.pdf]

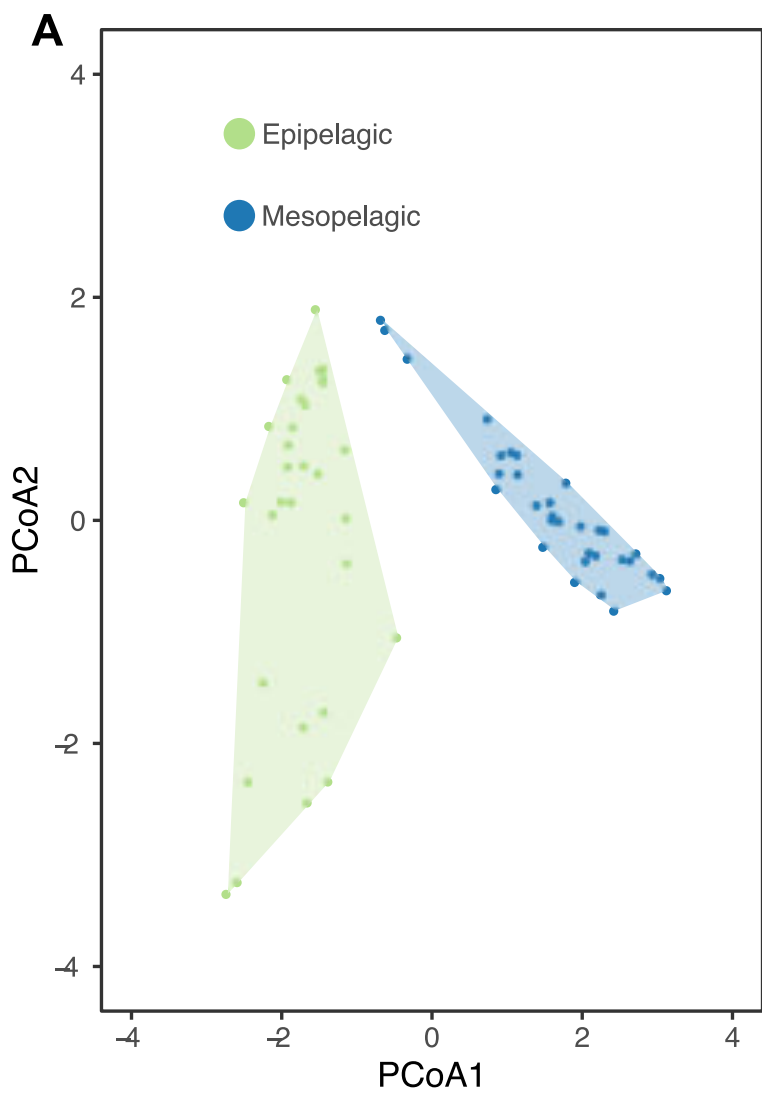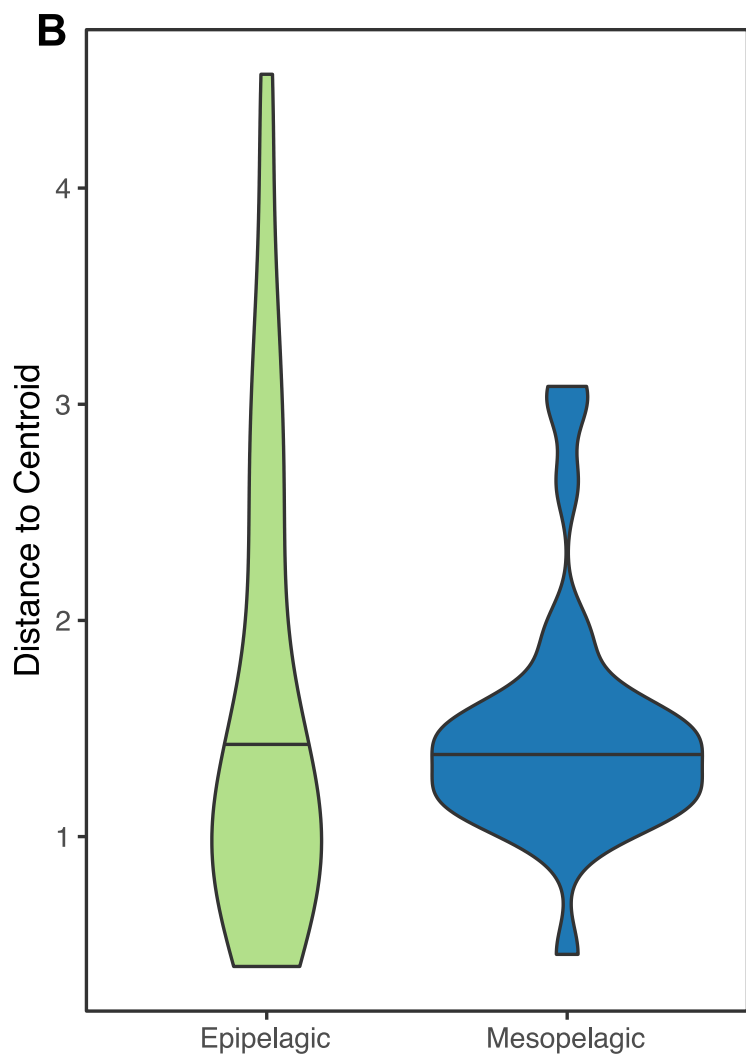

Supplement: Supplementary file 3 — Supplementary Figure S2 [file 43705_2023_279_MOESM3_ESM.pdf]

CLR Abundance

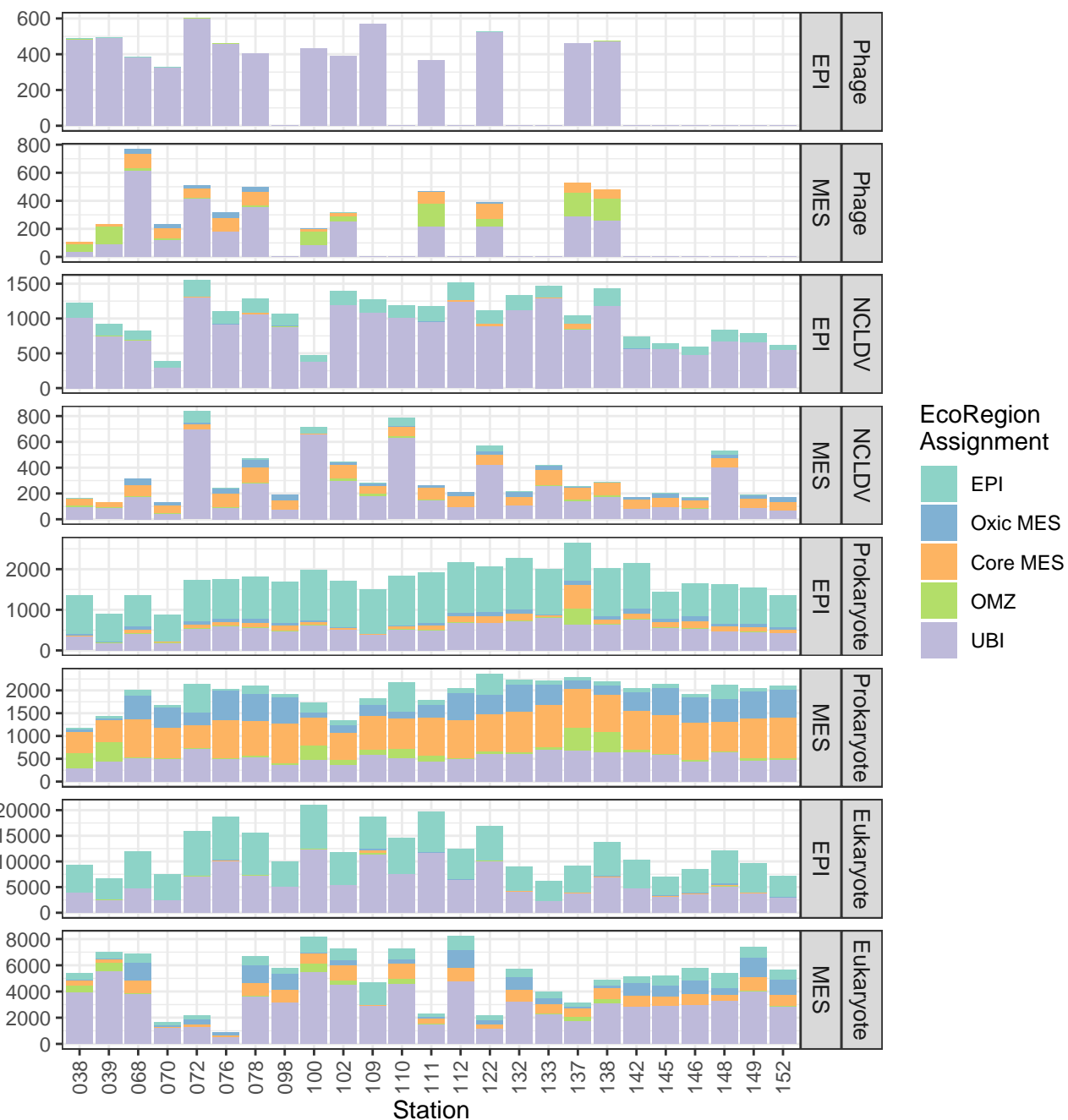

Supplement: Supplementary file 4 — Supplementary Figure S3 [file 43705_2023_279_MOESM4_ESM.pdf]

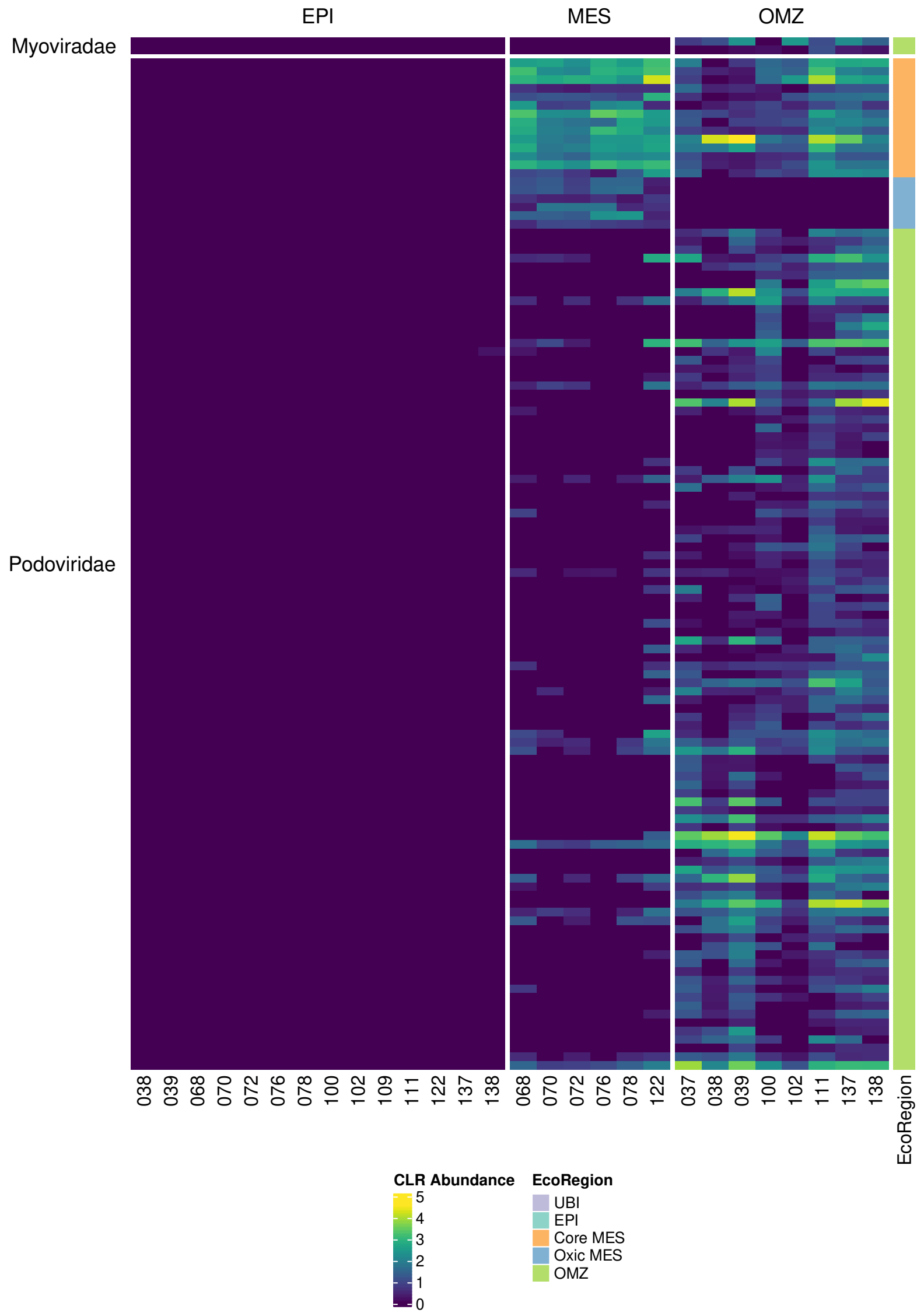

Supplement: Supplementary file 5 — Supplementary Figure S4 [file 43705_2023_279_MOESM5_ESM.pdf]

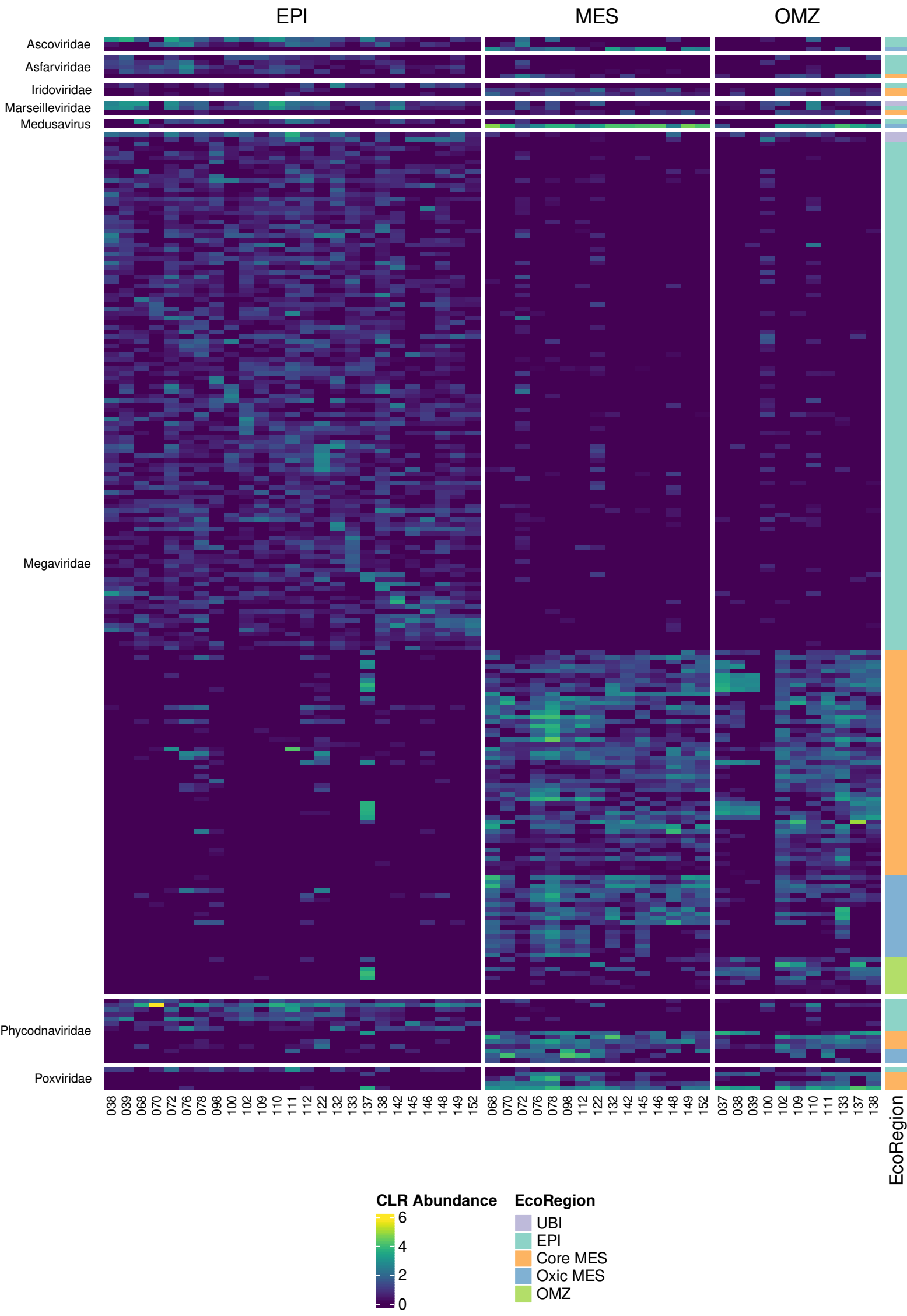

Supplement: Supplementary file 6 — Supplementary Figure S5 [file 43705_2023_279_MOESM6_ESM.pdf]

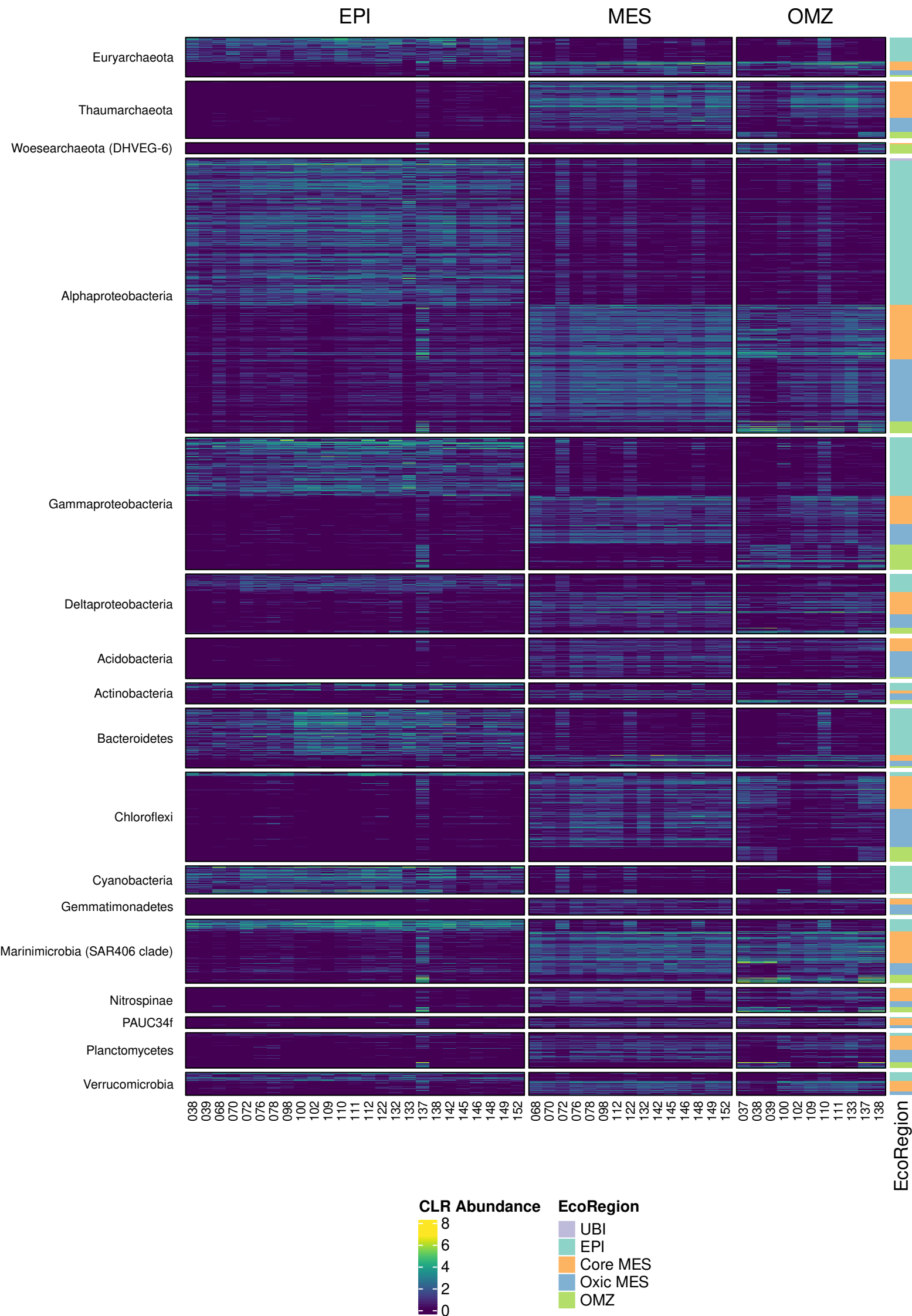

Supplement: Supplementary file 7 — Supplementary Figure S6 [file 43705_2023_279_MOESM7_ESM.pdf]

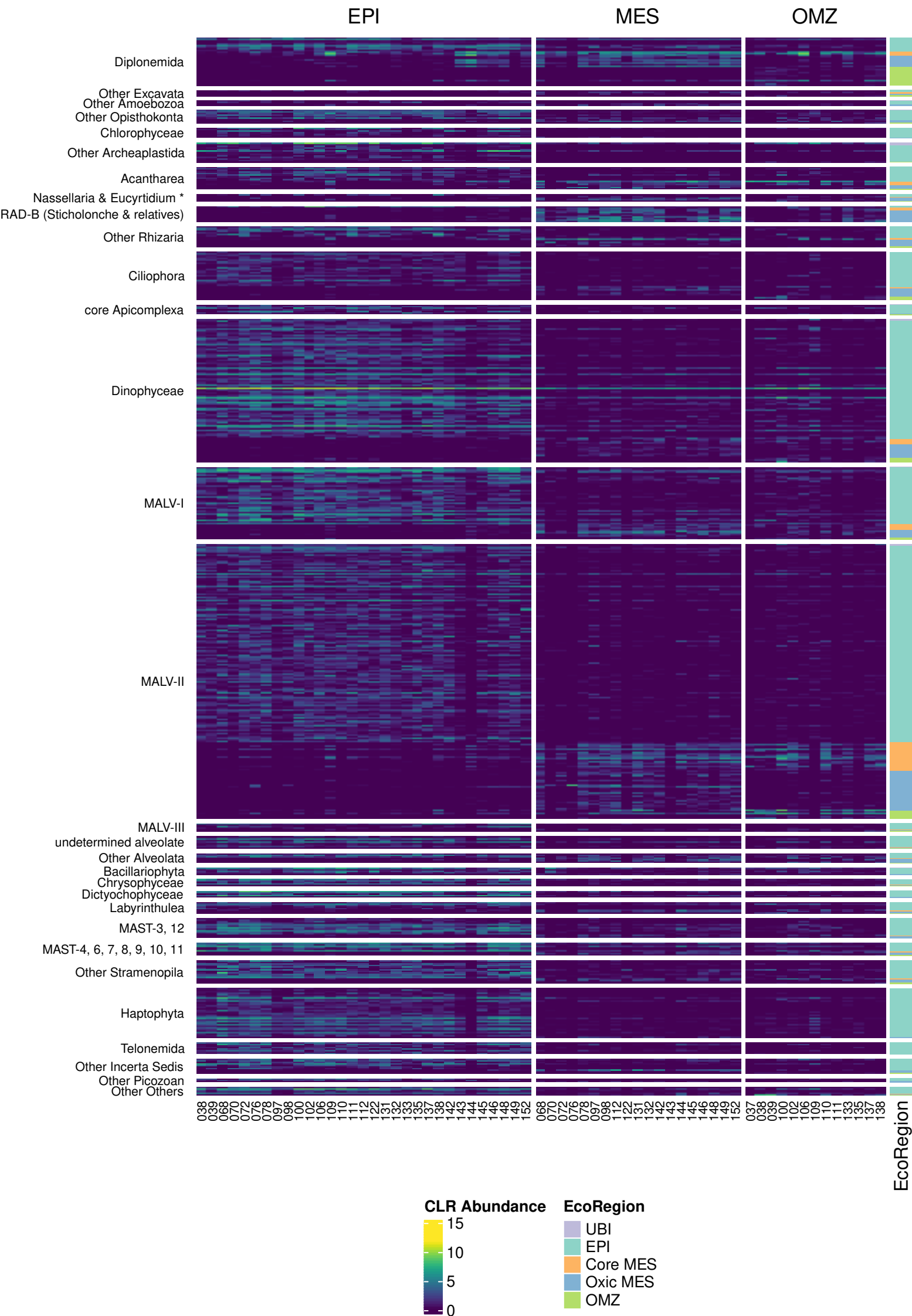

Supplement: Supplementary file 8 — Supplementary Figure S7 [file 43705_2023_279_MOESM8_ESM.pdf]
